# Bubble Point Measurements of *cis*-1,1,1,4,4,4-Hexafluorobutene [R-1336mzz(Z)] + *trans*-1,2-Dichloroethene [R-1130(E)] mixtures

**DOI:** 10.1007/s10765-024-03388-2

**Published:** 2024

**Authors:** Aaron J. Rowane, Stephanie L. Outcalt

**Affiliations:** 1 Material Measurement Laboratory, Applied Chemicals and Materials Division, National Institute of Standards and Technology, 325 Broadway, Boulder, CO 80305-3337, USA

**Keywords:** Bubble point, Refrigerant, Vapor–liquid equilibria, PC-SAFT

## Abstract

Saturation pressures of pure R-1336mzz(Z) and R-1130(E) and bubble point pressures of three R-1336mzz(Z)/1130(E) blends were measured from 265 K to 360 K. For each pure refrigerant or refrigerant blend, a total of twenty unique saturation pressures or bubble points were measured. In total 100 unique state points were obtained. Presently, no Helmholtz-energy-explicit type equation of state (EoS) is available for R-1130(E). While an extended corresponding states EoS for R-1130(E) is available to estimate the properties of the R-1336mzz(Z)/1130(E) blend, this model does not resolve the azeotropic behavior of the mixture. Therefore, the perturbed-chain statistical-associating fluid theory (PC-SAFT) EoS is used to model the vapor–liquid equilibria of the R-1336mzz(Z)/1130(E) blend. PC-SAFT model parameters are reported, and the overall performance of the model is characterized by deviations from the experimental data.

## 1 Introduction

*cis*-1,1,1,4,4,4-Hexafluorobutene (R-1336mzz(Z)) and *trans*-1,2-dichloroethene (R-1130(E)) are the two refrigerant components composing the refrigerant blend R-514A in ANSI/ASHRAE Standard 34 [1]. R-514A is a suitable replacement for the high global warming potential (GWP) refrigerant 2,2-dichloro-1,1,1-trifluoroethane (R-123) used in low-pressure centrifugal chillers. The benefits of R-514A over R-123 are that it offers comparable performance to R-123, a 97% reduction in GWP, and the blend forms an azeotrope meaning the blend has zero temperature glide. The R-514A blend is composed of 74.7% mass R-1336mzz(Z) with the balance being R-1130(E).

The optimal use of refrigerant blends containing R-1336mzz(Z) and R-1130(E) is contingent on the availability of thermodynamic data for each pure component in the mixture and blends at multiple compositions. These thermodynamic data can be used to develop reference quality mixture models to reproduce vapor pressures, densities, and heat capacities, which are relevant quantities used when designing refrigeration equipment. An abundance of pure R-1336mzz(Z) [2–10] and R-1130(E) [11–29] vapor pressure data is available with studies dating back as far as 1949 [2] and 1913 [11] respectively. Presently, phase equilibria data for the R-1336mzz(Z)/1130(E) blend is extremely limited, which hinders the development of reference quality mixture models. Only a single vapor–liquid equilibrium data set at 323.15 K included in Chemours application to the ANSI/ASHRAE standard-34 [1] is available for comparison. The present study reports measured vapor pressures of R-1336mzz(Z) and R-1130(E) and bubble points measured at three compositions ranging from (0.466 to 0.867) mass fraction of R-1336mzz(Z) at temperatures ranging from 265 K to 360 K.

An accurate reference EOS for R-1336mzz(Z) was developed by McLinden and Akasaka [9] and is available for use in REFPROP v10.0 [30]. The R-1336mzz(Z) is supported by high quality vapor pressure, saturated vapor, and liquid density, *pρT*, and sound speed data. However, there is a paucity of similar data for R-1130(E), for which only vapor pressure and saturated vapor and liquid density, and compressed liquid density data exist. Presently, the development of a reference EOS for R-1130(E) is premature given the scarcity of available thermodynamic data. Thus Tanaka et al. [28] developed an interim extended corresponding states model for the fluid, which can be used to estimate R-1130(E) thermodynamic properties. Preliminary calculations using the R-1336mzz(Z) reference EOS and the R-1130(E) ECS model with binary interaction parameters estimated using the method of Bell and Lemmon [31] did not predict the azeotropic behavior of this fluid. Therefore, in this study mixture modeling was done using the PC-SAFT EOS [32]. While the vapor pressure calculated with the PC-SAFT EOS were not of the accuracy expected of a reference-quality mixture EOS, they more accurately represented the mixture phase behavior compared to a mixture model incorporating the ECS model for R-1130(E).

## 2 Experimental

The apparatus, procedures and uncertainty analysis utilized in this work are much the same as those detailed by Outcalt and Rowane [33]. Thus, only brief descriptions are given here.

### 2.1 Mixture Preparation

The R-1336mzz(Z) and R-1130(E) samples used to prepare the mixtures evaluated in this study were obtained from commercial sources. Table 1 lists the CAS numbers, sample manufacturers, and purities. The purity listed for R-1336mzz(Z) is both the value provided by the manufacturer and that confirmed in-house from gas-chromatography/quadrupole time-of-flight mass spectroscopy (GC/QToF-MS) [9]. The purity for R-1130(E) was determined at NIST using nuclear magnetic resonance (NMR) spectroscopy. A detailed description of the NMR procedure is included in the supplemental information (SI).

Liquid phase mixtures were prepared gravimetrically with a balance having 0.1 mg resolution. Weighings during preparation were repeated four times with standard deviations of the weighings no greater than 0.02 g. Approximately, 388 g, 607 g and 390 g were prepared for the mixtures of R-1336mzz(Z) mass fractions 0.466, 0.748 and 0.867, respectively. Both pure compounds were degassed using a freeze/pump/thaw process prior to preparing mixtures to remove volatile impurities. Given the negligible uncertainty from gravimetric preparation the uncertainty contribution from the pure component impurities dominates the composition uncertainty. The composition uncertainties range from 0.001 to 0.002 mass fraction.

### 2.2 Apparatus

The vapor–liquid equilibria apparatus employed in this study has been described in detail in our previous studies and therefore the main features are only briefly summarized here. A more complete description of the apparatus can be found in our previous studies [33, 34] and in the SI. The apparatus consisted of a cylindrical, type-316 stainless-steel pressure vessel with opposing sapphire windows. The sapphire windows provided visual access of the cell’s contents to monitor both the liquid level and bubble size. Sample agitation to eliminate potential composition and temperature gradients in the sample was achieved with a stir bar located inside the pressure vessel and an external driving magnet. The cell and ancillary valves and tubing were housed inside a custom thermostat consisting of an insulated aluminum block with cartridge heaters and flow channels for heat transfer fluid to heat and cool the system, respectively. The operating conditions of the system range from 265 K to 360 K up to pressures of 7 MPa. The pressure vessel is connected to two oscillating quartz-crystal pressure transducers with maximum operating pressures of 0.7 MPa and 7 MPa. Both pressure transducers were held at a constant temperature of approximately 313 K and were calibrated against a piston gauge prior to making measurements. The temperature was measured using a standard platinum resistance thermometer (SPRT) located next to the measuring cell. The SPRT was regularly compared to temperature standards including the water triple point (273.16 K) and indium freezing point (429.749 K). Custom control code was used to fully automate the temperature control of the instrument, which is typically maintained within ± 0.005 K of the setpoint.

### 2.3 Measurement Procedure

Each mixture sample was contained within a stainless-steel sample cylinder located above the system and connected to the system with stainless-steel tubing. Prior to loading any sample, the entire internal volume of the system was evacuated. Any pressure transducer zero offset was recorded and subtracted from the reported pressures. To account for any shifts in the pressure transducer calibration the offset was also compared to those from previous runs. The value of the zero offsets changed no more than 0.04 kPa over the course of the measurements presented here. Samples were loaded such that a distinct liquid and vapor phase were present at the start of a constant composition (isopleth) run, but care was taken to ensure the size of the vapor bubble was very small relative to the liquid phase. Each isopleth run started at temperature of 265 K and was increased in increments of 5 K up to 360 K. As the temperature increased the vapor bubble began to disappear. To maintain a distinct vapor phase a small amount of liquid was periodically vented from the bottom of the cell through a pneumatic valve. At the end of an isopleth run, fresh sample was added to the residual sample from the previous run to determine whether venting sample resulted in a composition shift significant enough to change the bubble point pressure. The assumptions with this method of measurement are that the composition of the liquid phase is that of the bulk composition of the sample that was prepared gravimetrically, and that by loading the cell almost full of liquid with only a very small vapor space remaining, the pressure of the vapor phase is the bubble point pressure of the liquid composition at a given temperature.

### 2.4 Data and Uncertainty Analysis

Each bubble point pressure reported is the average of twenty measurements made at 30 s intervals at a given temperature. The standard deviation of the 20 pressure measurements was used as the repeatability of the pressure in the uncertainty analysis.

The line connecting the cell and pressure transducers runs up, out the top of the cell and thermostatted portion of the apparatus. It is exposed to ambient temperature prior to reaching the pressure transducers. The pressure transducers sit approximately 5 cm below the top of that line. Thus, at cell temperatures above ambient the line may become liquid filled and a hydrostatic pressure correction is applied. This correction is made using REFPROP [30] to predict the saturated liquid density of the sample at 294 K (approximately ambient temperature) and using the formula $\Delta P=\rho_{\text{sat}\cdot }g\cdot h$, where $g=9.81\;m/s^{2}$ and h=0.05m m to calculate the pressure correction. The pressure resulting from this calculation is subtracted from the measured pressure at temperatures above 295 K. Saturated liquid densities for the mixtures were calculated by multiplying the mole fraction of the pure components by their pure component saturated liquid density and summing the two.

Four main sources of uncertainty were considered in this work: temperature, pressure, sample composition and measurement repeatability. Through regular calibration checks (against the triple point of water and the freezing point of indium), the uncertainty in temperature as measured with the standard platinum resistance thermometer (SPRT) was 0.02 K.

The uncertainty in the pressure measurement consisted of the repeatability of the measurement over the 20 readings that were averaged for each reported point and the accuracy of the pressure transducer. The manufacturers’ stated accuracy of the pressure transducers is 0.01% of full scale or 0.07 kPa and 0.7 kPa for the low- and high-range transducers, respectively. Recent calibration checks against a piston-gauge pressure standard showed deviations from the standard of no more than 0.02 kPa for the low-pressure gauge and 0.09 kPa for the high-range gauge. As the lower-pressure gauge covered the entire pressure range of this work and its accuracy was better, we report only the data from that instrument and assign a standard uncertainty of 0.035 kPa (half the manufacturer’s stated uncertainty) to the pressure readings from that gauge.

The mixtures measured in this work were prepared gravimetrically following the procedure detailed in Outcalt and Rowane [33]. As stated previously the largest standard deviation of the repeated weighings during mixture preparation was 0.02 g. Given the large amount of sample prepared this would have negligible impact on the stated compositions. Thus, the largest uncertainty in the composition of the samples as prepared with this method is from the possible incomplete evacuation of entrained air. While preparation of the samples (both pure and mixtures) included several freeze/pump/thaw cycles, residual air contamination must be considered. We assigned a value of 0.1 kPa to this and considered it a constant contribution to the combined standard uncertainty. A complete description of how this number was derived is given in the SI as “Calculation of Incomplete Evacuation of Samples.”

The relatively high normal boiling points (R-1336mzz(Z) *T*_nbp_ ~ 306.6 K, R-1130(E) *T*_nbp_ ~ 321.7 K) of the refrigerants and blends measured in this study result in low bubble point pressures and mean that even small uncertainties in pressure equate to large percentage uncertainties. The combined standard uncertainty of the reported bubble point measurements was calculated with the root sum of squares method as found in the Guidelines for Evaluating and Expressing the Uncertainty of NIST Measurement Results [35]. The sources of uncertainty and their ranges are given in Table 2. The reported uncertainties in Table 3 have a coverage factor of *k* = 2.

## 3 Results

### 3.1 Experimental Data

Saturation pressures of R-1336mzz(Z) and R-1130(E) and bubble points of the R-1336mzz(Z)/1130(E) mixture are listed in Table 3. The combined expanded uncertainty is reported as both an absolute value in pressure and the percentage of the measured bubble point pressure. The data reported in this paper can also be found at data.nist.gov (https://doi.org/10.18434/mds2-3279) in machine readable format.

### 3.2 Comparison to REFPROP

McLinden and Akasaka [9] developed a robust EOS for R-1336mzz(Z), which was fit to available saturation, density, and speed of sound data. Tanaka et al. [28] developed an interim extended corresponding state (ECS) model for R-1130(E) and included a.FLD file for use with REFPROP[30]. Presently, insufficient data are available to develop a robust reference EOS for R-1130(E). Table 4 summarizes comparisons of the present experimental saturation pressures to those calculated using the models implemented in REFPROP[30] with the absolute average deviation $\left(\Delta_{\text{AAD}}\right)$, bias $\left(\Delta_{\text{bias}}\right)$, and maximum deviation $\left(\Delta_{max}\right)$ given by,

(1) $$\Delta_{\text{AAD}}=100\cdot \frac{1}{N}\sum_{i=1}^{N}\frac{\left|p_{i,\text{exp}}\;-\;p_{i,\text{calc}}\right|}{p_{i,\text{calc}}}$$

(2) $$\Delta_{bias}=100\cdot \frac{1}{N}\sum_{i=1}^{N}\frac{p_{i,\exp}\;-\;p_{i,\text{calc}}}{p_{i,\text{calc}}}$$

(3) $$\Delta_{max}=max\left(100\cdot \frac{\left|p_{i,\text{exp}}\;-\;p_{i,\text{calc}}\right|}{p_{i,\text{calc}}}\right),$$

where $p_{i,\text{exp}}$ is an experimentally determined saturation pressure, and $p_{i,\text{calc}}$ is a saturation pressure calculated using models implemented in REFPROP[30]. Comparisons are limited to studies reporting three or more datapoints.

**Fig. 1:**
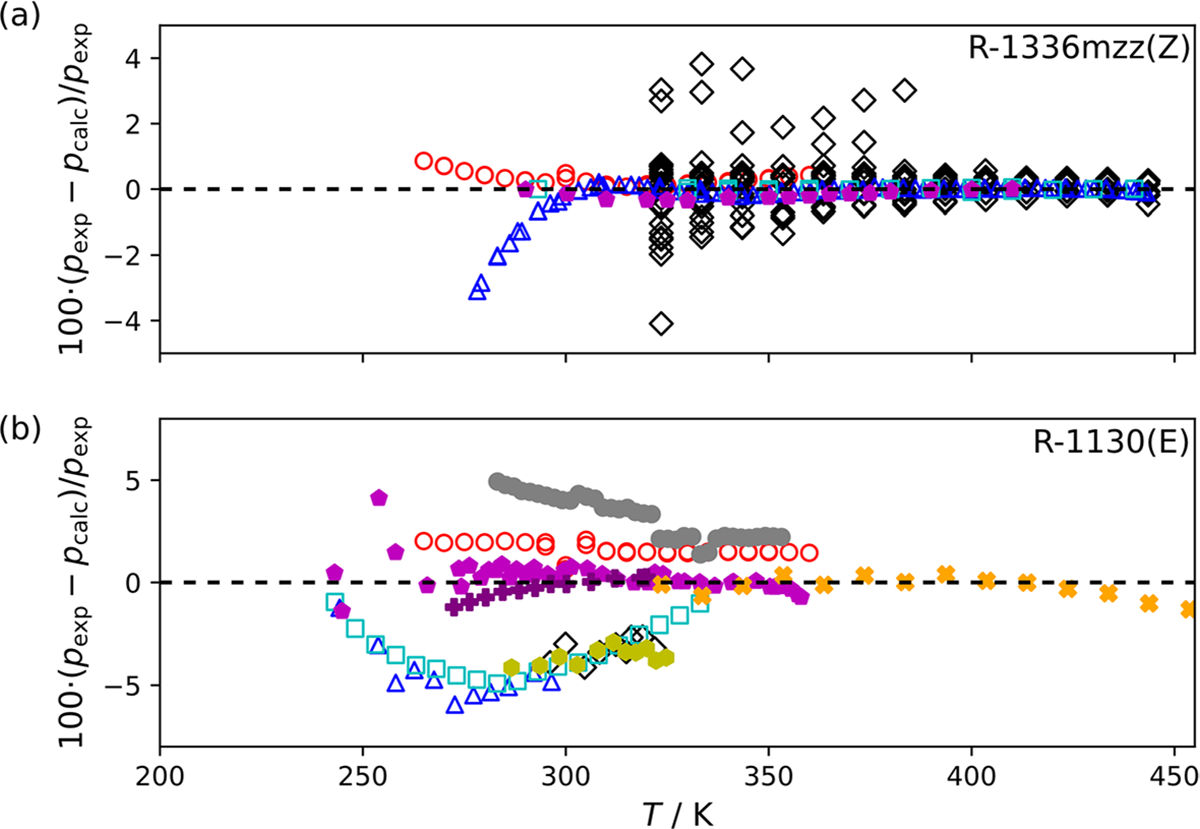
Comparison of experimentally measured vapor pressures, $p_{\text{exp}}$, to calculated vapor pressures, $p_{\text{calc}}$, with (a) the EOS of McLinden and Akasaka for R-1336mzz(Z): 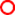
—this study, 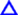
—Li et al. [8], 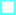
—McLinden and Akasaka[9], 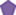
—Sakoda et al. [10], 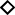
—Tanaka et al. [7], and (b) an extended corresponding states model of Tanaka et al. for R-1130(E): 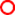
—this study, 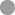
—Lombardo et al. [29], 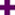
—Tanaka et al. [28], 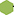
—Machat and Boublik [23], 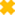
—Flom et al. [18], 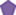
—Ketelaar et al. [16], 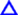
—Hsia [12], 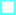
—Hsia [13], and 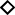
—Herz and Rathmann [11] (Color figure online)

Figure 1a and b are deviation plots comparing experimentally measured saturation pressures from this study and the available literature data to those calculated with equations of state implemented in REFPROP[30] for R-1336mzz(Z) and R-1130(E), respectively. In Fig. 1a the data of Tanaka et al. [7] show significant scatter between 323 K to 444 K relative to the other studies reporting vapor pressures for R-1336mzz(Z). The scatter in the data of Tanaka et al. [7] is not surprising given that they were not direct vapor pressure measurements and were derived from density measurements in the two-phase region. At temperatures below 300 K the data of Li et al. [8] show consistent negative deviations relative to the reference EOS. However, above 300 K, excluding the scatter in the data of Tanaka et al. [7], the data reported in this study, Li et al. [8], McLinden and Akasaka [9], and Sakoda et al. [10] compare within ± 2%. In Fig. 1b saturation pressures for R-1130(E) reported by Machat and Boublik [23], Hsia [12, 13], and Herz and Rathmann [11] are consistently negative relative to the ECS model from 250 K to 340 K. It is interesting to note there was a specific focus in these studies to remove the higher boiling-point (less volatile) stereoisomer R-1130(Z) from their sample. While the procedure used to purify their sample was provided, an exact sample purity was not stated. Residual R-1130(Z) in the R-1130(E) sample used by these studies is a reasonable explanation for the consistent negative deviations since the addition of the less volatile R-1130(Z) stereoisomer is expected to result in lower saturation pressures. The remaining studies have generally consistent agreement in comparison to the R-1130(E) ECS model. Although it should be noted that the saturation pressures for both R-1336mzz(Z) and R-1130(E) reported in this study show a consistent slight positive deviation relative to the models implemented in REFPROP [30].

In this study only pure R-1336mzz(Z) and R-1130(E) saturation data are compared to REFPROP [30] models. Vapor–liquid equilibria calculations incorporating the R-1336mzz(Z) reference EOS and R-1130(E) ECS model with estimated binary interaction parameters do not resolve the azeotropic nature of the blend. Thus, we compare the mixture data from the present study to the PC-SAFT EOS.

### 3.3 PC-SAFT EOS Modeling

Saturation pressures of pure R-1336mzz(Z) and R-1130(E) and bubble point pressures of the R-1336mzz(Z)/1130(E) blends are modeled using the Perturbed-Chain Statistical-Associating Fluid Theory (PC-SAFT) equation of state (EOS). Details of the PC-SAFT EOS are described by Gross and Sadowski [32] and additional details can be found in the supporting information (SI). Only the estimation scheme for the model parameters and mixing rules are restated here. All calculations were carried out using the open-source thermodynamics package teqp [36]. The PC-SAFT EOS has three parameters for each component, which are m, the number of segments per chain molecule, σ, the segment diameter, and $\varepsilon /k_{b}$, the depth pair potential well-divided by the Boltzmann constant $k_{b}$. As done in our previous study [34], m,σ, and $\varepsilon /k_{b}$ are estimated using the correlation developed by Anoune et al. [37], given by Eqs. 4 to 6, which were found to provide more reliable vapor–liquid equilibrium and critical point calculations for non-associating compounds. Using this methodology, the required inputs for Eqs. 4 to 6 are the critical temperature, $T_{c}$, critical pressure, $p_{c}$, and acentric factor, ω. PC-SAFT parameters used in this study are listed in Table 5 along with each pure component’s critical temperature, critical pressure, acentric factor, and molar mass.


(4) $$m=0.43344\omega^{2}+7.84968\omega +0.92734$$

(5) $$\sigma^{3}\left(\frac{p_{c}}{T_{c}}\right)=-0.06388{\left(\frac{1}{m}\right)}^{2}+1.28018\left(\frac{1}{m}\right)-0.03879$$

(6) $$\left(\frac{\varepsilon }{k_{b}}\right)/T_{c}=-0.15924{\left(\frac{1}{m}\right)}^{2}+0.70433\left(\frac{1}{m}\right)+0.24264$$

**Fig. 2:**
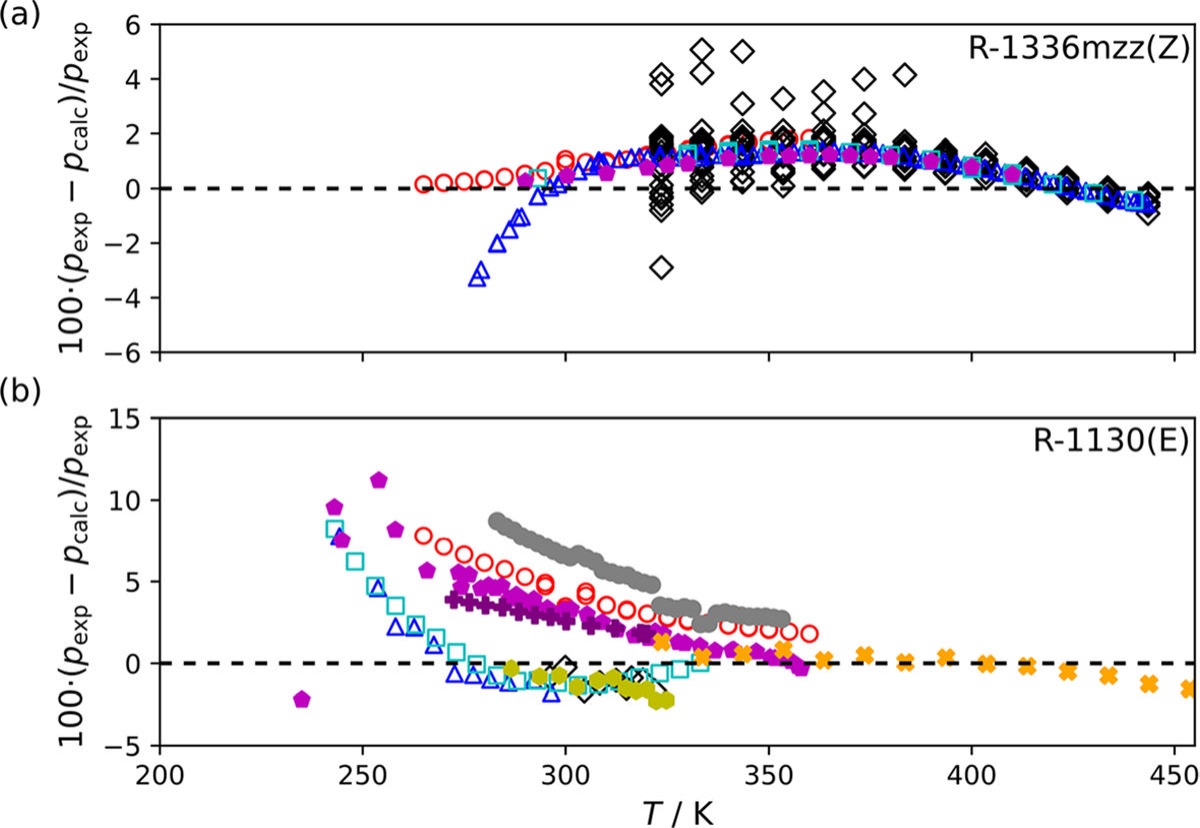
Comparison of experimentally measured vapor pressures, $p_{\text{exp}}$, to calculated vapor pressures, $p_{\text{calc}}$, with the PC-SAFT EOS using model parameters calculated using Eqs. 1 to 3 for (a) R-1336mzz(Z): 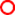
—this study, 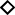
—Tanaka et al. [7], 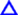
—Li et al. [8], 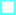
—McLinden and Akasaka [9], 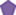
—Sakoda et al. [10], and (b) R-1130(E): 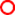
—this study, 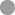
—Lombardo et al. [29], 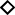
—Herz and Rathmann [11], 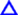
—Hsia [12], 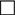
—Hsia[13], 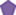
—Ketelaar et al.[16], 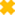
—Flom et al .[18], 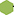
—Machat and Boublik [23], and 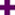
—Tanaka et al. [28] (Color figure online)

Figure 2 compares experimental vapor pressures from this study and those available in the literature for R-1336mzz(Z) and R-1130(E) to those calculated with the PC-SAFT EOS using parameters estimated from Eqs. 4 to 6. Again, the comparisons in Fig. 2 are limited to studies reporting 3 or more data points. The deviations shown in Fig. 2a and b are unsurprisingly greater than those seen in Fig. 1a and b given the predictive framework of this modeling technique. Table 6 is similar in content to Table 4 but showing comparisons to the PC-SAFT EOS with model parameters calculated using Eqs. 4 to 6 for R-1336mzz(Z) and R-1130(E).

**Fig. 3:**
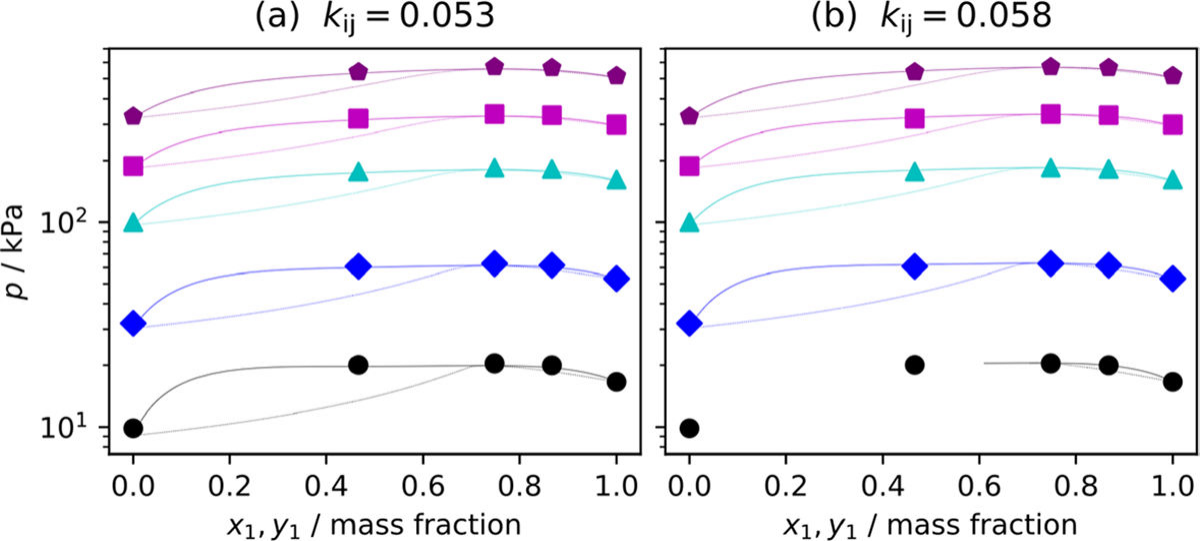
p−xy traces of R-1336mzz(Z)/1130(E) binary mixture using the PC-SAFT EoS with (a) binary interaction parameter selected to suppress LLE at T < 280 K and (b) selected to be the best fit of the data at 320 K. Traces are shown at isotherms of 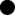
—265 K, 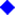
—290 K, 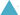
—320 K, 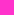
—340 K, and 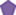
—360 K (Color figure online)

**Fig. 4:**
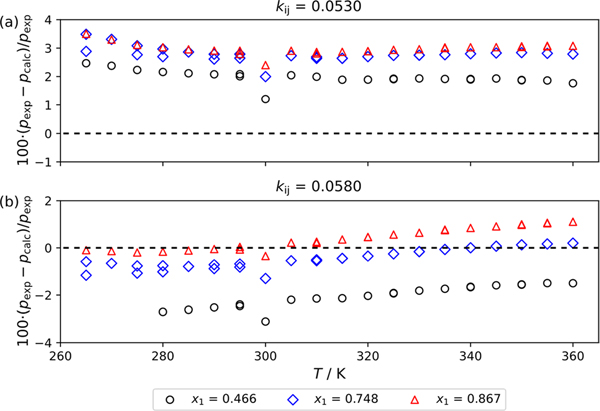
Deviations between experimentally measured bubble point pressures reported in this study, $p_{\text{exp}}$, and those calculated with the PC-SAFT EOS, $p_{\text{exp}}$, where (a) $k_{ij}=0.0530$ to avoid calculations that show LLE and (b) $k_{ij}=0.0580$ which was obtained from the best fit of the 320 K isotherm

Mixture parameters, $\varepsilon_{ij}$, and $\sigma_{ij}$ are defined by conventional mixing rules,

(7) $$\bar{m}=\sum_{i}x_{i}m_{i}$$

(8) $$\sigma_{ij}=\frac{\left(\sigma_{i}\;+\;\sigma_{j}\right)}{2}$$

(9) $$\varepsilon_{ij}=\left(1-k_{ij}\right)\sqrt{\varepsilon_{i}\varepsilon_{j}},$$

where $k_{ij}$ is the binary interaction parameter typically determined from the best fit of the data. The $k_{ij}$ value was obtained by minimizing the sum of the absolute residuals in pressure $\left(\Sigma \left|p_{\text{exp}}-p_{calc}\right|\right)$ for a single isotherm, which was chosen to be 320 K. The $k_{ij}$ obtained from the best fit of the data were found to be 0.058. However, for this mixture, the PC-SAFT model predicted the presence of liquid–liquid equilibrium at temperatures below 280 K. Through trial and error, it was found that limiting $k_{ij}$ values to a maximum of 0.053 eliminated LLE over the range of conditions investigated in this study. In our investigation LLE was not observed. Therefore, results from two $k_{ij}$ values are provided and evaluated. Figure 3a and b show several p−x traces using the PC-SAFT EOS with $k_{ij}=0.053$ and $k_{ij}=0.058$, respectively. In Fig. 3a, where a $k_{ij}$ value of 0.053 is used, the p−x traces at all temperatures ranging from 265 K to 360 K are continuous across all compositions investigated. However, in Fig. 3b the 265 K isotherm is discontinuous at $x_{1}<0.5$ as the model predicts a vapor–liquid–liquid equilibrium (VLLE) region. Figure 4a and b are deviation graphs comparing the experimental data reported in this study to those calculated using the PC-SAFT EOS with $k_{ij}$ values of 0.053 and 0.058, respectively. Figure 4(a) shows that most of the data consistently deviate from the PC-SAFT EOS between 2 to 3%. Figure 4(b) shows more favorable comparison for compositions of *x* = (0.748 to 0.867) mass fraction. Here the experimental data and model agree within ± 2%. However, where x=0.466 mass fraction where the model anticipates a vapor–liquid–liquid equilibrium (VLLE) the model is unable to reproduce the VLE observed at temperatures below 280 K in our experiments and deviations exceed 3%. Of note in Fig. 4a and b is the discontinuity in the data at approximately 300 K. This is the temperature region where the line connecting the top of the cell and the pressure transducers starts to fill with liquid as the cell temperature becomes greater than ambient. We have attempted a correction to our data with the hydrostatic head correction but, quantifying the height of the head as it forms is difficult.

**Fig. 5:**
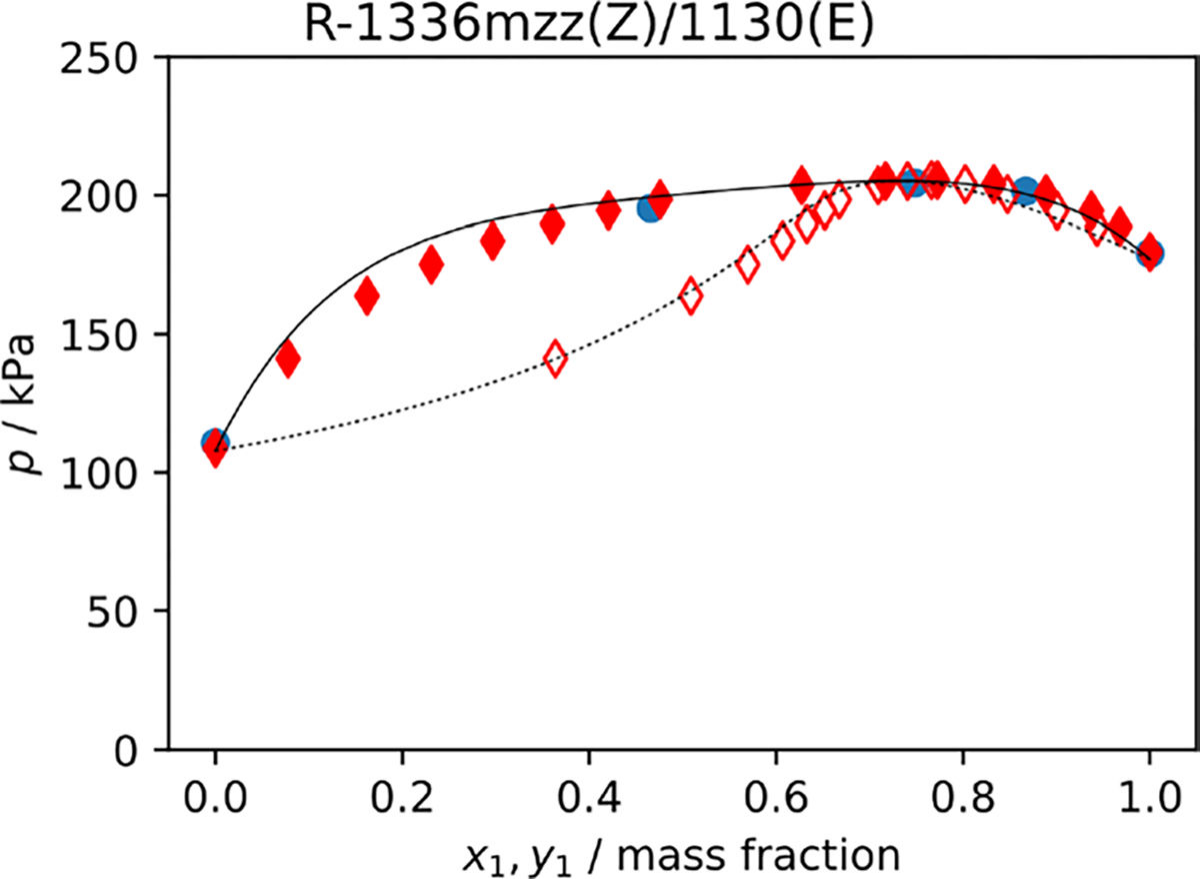
Pressure as a function of composition at 323.15 K for the R-1336mzz(Z)/1130(E) blend. Bubble and dew points are represented by filled and unfilled symbols, respectively. Lines drawn are calculations with the PC-SAFT EOS with $k_{ij}=0.0588$ where solid lines represent the bubble line and dashed lines represent the dew line. This study ((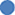)) and included in ASHRAE std 34 submission [1] ((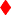,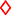))

Only a single experimental VLE data set for the R-1336mzz(Z)/1130(E) mixture reported in the ASHRAE application for R-514A [1] is available for comparison. The data set reported in the ASHRAE application is extremely limited as it only reports a single *p*-*xy* loop at 323.15 K. Figure 5 is a p−xy plot of the data reported in the ASHRAE application and those obtained through linear interpolation of the data reported in the present study. Also shown in the plot is the PC-SAFT EOS with $k_{ij}$ set to 0.058. Figure 5 shows that the bubble point pressures interpolated from the data in present study and those reported in the ASHRAE application are consistent.

## 4 Discussion and Conclusions

Vapor pressures are reported for the pure fluids R-1336mzz(Z) and R-1130(E), and bubble point pressures for the R-1336mzz(Z)/1130(E) mixture are reported at three compositions. The pure fluid data reported in this study, along with available literature data were compared to the EOS of McLinden and Akasaka [9] for R-1336mzz(Z) and the EOS of Tanaka et al. [28] for R-1130(E). The vapor pressure data for both R-1336mzz(Z) and R-1130(E) presented in this study are within the scatter of the available literature data. The bubble point data reported for the binary mixtures represent a large increase in such data as only one other limited data set currently exist in the literature. These data were modeled using the PC-SAFT EOS with parameters estimated using critical property data. Modeling vapor pressures using the predictive PC-SAFT approach unsurprisingly resulted in significantly larger deviations from the experimental data than the equations reported by McLinden and Akasaka [9] and Tanaka et al. [28] However, the PC-SAFT EOS could resolve the azeotropic nature of the R-1336mzz(Z)/1130(E) mixture unlike the mixture modeling approach incorporating the EOS of McLinden and Akasaka [9] and ECS model of Tanaka et al. [28] with estimated binary interaction parameters.

## Supplementary Material

Supp1

## Figures and Tables

**Table 1:** Suppliers of pure fluids used in the preparation of mixtures for this work

Chemical	CAS #	Manufacturer	Analysis method	Purity [mole frac]

*cis*-1,1,1,4,4,4-hexafluorobut-2-ene (R-1336mzz(Z))	692-49-9	Chemours	GC/QToF-MS^a^	0.9999
*trans*-1,2-dichloroethane (R-1130(E))	156-60-5	Chemours	NMR^b^	>0.9910

a Gas-chromatography/quadrupole time-of-flight mass spectroscopy

b Nuclear magnetic resonance

**Table 2:** Sources of uncertainty included in the combined standard uncertainty

Property	Uncertainty (*k* = 1)	Equivalent in pressure [kPa]

Temperature measurement	0.02 K	0.01–0.26
Pressure transducer measurement		0.035
Repeatability of measurements		0.000–0.260
Incomplete vacuum		0.1
Total Root Sum of Squares (*k* = 1)		0.11–0.38

Ranges include the maximum and minimum values for the samples reported in this work

**Table 3:** Experimental bubble point data measured in this study for the R-1336mzz(Z)/1130(E) blend, where *x*_1_ corresponds to the mass fraction of R-1336mzz(Z). Listed are the temperature (*T*), pressure (*p*), combined expanded uncertainty in pressure, with a coverage factor of *k* = 2, (*U*_c_(*p*)), and the percent uncertainty (100·*U*_c_(*p*)/*p*). The mass fraction standard uncertainty (*u*_c_(*x*_1_)) is listed next to each composition

*T*/K	*p*/kPa	*U*_c_(*p*)/kPa	100·*U*_c_(*p*)/*p*

*x*_1_ = 0.000 (Pure R-1130(E))			
265.00	9.86	0.22	2.23
270.00	12.73	0.22	1.73
275.00	16.28	0.22	1.35
280.00	20.60	0.22	1.07
285.00	25.84	0.22	0.85
290.00	32.08	0.22	0.69
295.00	39.44	0.22	0.56
295.00	39.52	0.22	0.56
300.00	47.66	0.22	0.46
300.00	47.75	0.22	0.46
305.00	58.51	0.22	0.38
305.00	58.66	0.22	0.38
310.00	70.25	0.24	0.34
310.00	70.26	0.24	0.34
315.00	83.98	0.24	0.29
315.00	84.03	0.24	0.29
320.00	99.87	0.26	0.26
320.00	99.87	0.26	0.26
325.00	117.92	0.26	0.22
325.00	118.00	0.26	0.22
330.00	138.65	0.28	0.20
330.00	138.57	0.28	0.20
335.00	161.97	0.28	0.17
335.00	162.03	0.28	0.17
340.00	188.34	0.30	0.16
340.00	188.39	0.30	0.16
345.00	217.86	0.32	0.15
345.00	217.95	0.32	0.15
350.00	251.00	0.34	0.14
350.00	250.92	0.34	0.14
355.00	287.69	0.38	0.13
360.00	328.39	0.40	0.12
*x*_1_ = 0.466 (*u*_c_(*x_1_*) = 0.002)			
265.00	20.05	0.22	1.10
270.00	25.53	0.22	0.86
270.00	25.47	0.24	0.94
275.00	32.15	0.22	0.68
275.00	32.13	0.22	0.68
280.00	40.12	0.22	0.55
280.00	40.45	0.22	0.54
285.00	49.61	0.22	0.44
285.00	49.58	0.22	0.44
290.00	60.82	0.22	0.36
295.00	73.99	0.24	0.32
295.00	73.94	0.24	0.32
300.00	88.56	0.26	0.29
305.00	107.02	0.26	0.24
310.00	127.33	0.26	0.20
310.00	127.25	0.26	0.20
315.00	150.46	0.32	0.21
320.00	176.91	0.30	0.17
325.00	206.84	0.32	0.15
325.00	206.81	0.32	0.15
330.00	240.54	0.34	0.14
335.00	278.27	0.38	0.14
340.00	320.37	0.40	0.12
340.00	320.32	0.42	0.13
345.00	367.18	0.44	0.12
350.00	418.77	0.48	0.11
350.00	418.83	0.48	0.11
355.00	475.94	0.52	0.11
360.00	538.46	0.56	0.10
*x*_1_ = 0.748 (*u*_c_(*x_1_*) = 0.002)			
265.00	20.34	0.22	1.08
265.00	20.46	0.22	1.08
270.00	26.11	0.22	0.84
270.00	25.99	0.22	0.85
275.00	32.96	0.22	0.67
275.00	32.86	0.22	0.67
280.00	41.12	0.22	0.54
280.00	41.23	0.22	0.53
285.00	51.09	0.22	0.43
285.00	50.99	0.22	0.43
290.00	62.78	0.22	0.35
290.00	62.68	0.22	0.35
295.00	76.42	0.24	0.31
295.00	76.53	0.24	0.31
300.00	91.90	0.26	0.28
305.00	111.15	0.26	0.23
310.00	132.50	0.28	0.21
310.00	132.44	0.28	0.21
315.00	156.97	0.30	0.19
315.00	156.97	0.30	0.19
320.00	184.94	0.32	0.17
325.00	216.63	0.34	0.16
330.00	252.31	0.38	0.15
330.00	252.33	0.40	0.16
335.00	292.43	0.46	0.16
340.00	337.23	0.42	0.12
345.00	387.11	0.52	0.13
345.00	387.05	0.52	0.13
350.00	442.44	0.54	0.12
355.00	503.48	0.60	0.12
360.00	570.64	0.68	0.12
*x*_1_ = 0.867 (*u*_c_(*x_1_*) = 0.001)			
265.00	19.99	0.22	1.10
265.00	19.94	0.22	1.10
270.00	25.53	0.22	0.86
275.00	32.25	0.22	0.68
275.00	32.19	0.22	0.68
280.00	40.38	0.22	0.54
285.00	50.08	0.22	0.44
285.00	50.02	0.22	0.44
290.00	61.59	0.22	0.36
295.00	75.13	0.24	0.32
295.00	75.05	0.24	0.32
300.00	90.50	0.26	0.29
305.00	109.27	0.26	0.24
310.00	130.27	0.26	0.20
310.00	130.34	0.28	0.21
315.00	154.53	0.30	0.19
320.00	182.14	0.32	0.18
320.00	182.15	0.34	0.19
325.00	213.45	0.36	0.17
330.00	248.74	0.42	0.17
335.00	288.50	0.48	0.17
335.00	288.46	0.46	0.16
340.00	332.83	0.50	0.15
345.00	382.21	0.54	0.14
350.00	437.03	0.58	0.13
350.00	437.15	0.58	0.13
355.00	497.83	0.66	0.13
355.00	497.74	0.66	0.13
360.00	564.65	0.76	0.13
*x*_1_ = 1.000 (Pure R-1336mzz(Z))			
265.00	16.57	0.22	1.33
270.00	21.35	0.22	1.03
270.00	21.35	0.22	1.03
275.00	27.20	0.22	0.81
280.00	34.30	0.22	0.64
280.00	34.30	0.22	0.64
285.00	42.84	0.22	0.51
290.00	53.02	0.22	0.41
290.00	53.02	0.22	0.41
295.00	65.05	0.24	0.37
300.00	79.43	0.24	0.30
300.00	79.31	0.26	0.33
305.00	95.73	0.26	0.27
310.00	114.71	0.28	0.24
310.00	114.65	0.28	0.24
315.00	136.58	0.30	0.22
320.00	161.69	0.32	0.20
320.00	161.77	0.32	0.20
325.00	190.26	0.32	0.17
330.00	222.66	0.36	0.16
330.00	222.62	0.38	0.17
335.00	259.13	0.38	0.15
340.00	300.03	0.42	0.14
340.00	299.93	0.42	0.14
345.00	345.74	0.46	0.13
350.00	396.50	0.48	0.12
350.00	396.60	0.52	0.13
355.00	453.00	0.56	0.12
360.00	515.28	0.62	0.12
360.00	515.27	0.68	0.13

The standard uncertainty in temperature is 0.02 K

**Table 4:** Summary of model comparisons to R-1336mzz(Z) reference equation of McLinden and Akasaka and R-1130(E) extended corresponding states model of Tanaka et al.

Study	Year	*T*_range_/K	Purity	Δ_AAD_	Δ_bias_	Δ_max_

R-1336mzz(Z)						
This study	2024	265–360	0.9999	0.18	0.17	0.41
Li et al	2020	278–443	0.997	0.23	−0.19	0.21
McLinden and Akasaka	2020	293–440	0.9999	0.03	−0.01	0.05
Sakoda et al	2020	290–410	0.999	0.18	−0.18	0.01
Tanaka et al	2016	323–444	0.9995	0.57	0.15	3.82
R-1130(E)						
This study	2024	265–360	> 0.991	1.39	1.39	1.91
Lombardo et al	2023	283–353	0.997	3.19	3.19	4.93
Tanaka et al	2022	324–454	0.997	0.39	−0.22	0.40
Machat and Boublik	1985	272–320	0.991	0.40	−0.29	0.25
Flom et al	1951	287–325	–	3.58	−3.58	−2.90
Ketelaar et al	1947	235–358	–	1.11	0.27	13.83
Hsia	1931	244–296	–	4.47	−4.47	−1.24
Hsia	1931	243–333	–	3.32	−3.32	−0.95
Herz and Rathmann	1913	296–322	–	3.26	−3.26	−2.65

**Table 5:** PC-SAFT parameters segment length (*m*), segment diameter (σ), and segment energy (ε/*k*_b_) for R-1336mzz(Z) and R-1130(E) calculated using Eqs. 1 to 3 with inputs being the critical temperature (*T*_c_), critical pressure (*p*_c_), and acentric factor (*ω*)

Fluid	MW/g·mol^−1^	*T*_c_/K	*p*_c_/MPa	*ω*	*m*	*σ*/Å	*ε*/*k*_b_/K

R-1336mzz(Z)	164.06	444.5[7]	2.903[7]	0.386[9]	4.0219	3.4814	181.32
R-1130(E)	96.94	516.5[38]	5.510[38]	0.2137[28]	2.6246	3.4543	251.99

**Table 6:** Summary of comparison to the PC-SAFT EOS with parameters obtained from Eqs. 4 through 6

Study	Year	*T*_range_/K	Purity	Δ_AAD_	Δ_bias_	Δ_max_

R-1336mzz(Z)						
This study	2024	265–360	0.9999	0.97	0.90	1.81
Li et al	2020	278–443	0.997	0.97	0.57	1.40
McLinden and Akasaka	2020	293–440	0.9999	0.95	0.88	1.42
Sakoda et al	2020	290–410	0.999	0.91	0.91	1.23
Tanaka et al	2016	323–444	0.9995	1.24	1.13	5.07
R-1130(E)						
This study	2024	265–360	> 0.991	3.43	3.43	6.83
Lombardo et al	2023	283–353		4.98	4.98	8.71
Tanaka et al	2022	324–454	0.997	0.60	−0.01	1.30
Machat and Boublik	1985	272–320	0.991	2.85	2.85	3.89
Flom et al	1951	287–325	–	1.32	−1.32	−0.32
Ketelaar et al	1947	235–358	–	3.48	3.36	17.98
Hsia	1931	244–296	–	2.20	1.06	7.78
Hsia	1931	243–333	–	1.95	0.93	8.24
Herz and Rathmann	1913	296–322	–	1.11	−1.11	−0.24

## Data Availability

The data reported in this paper can also be found at data.nist.gov (https://doi.org/10.18434/mds2-3279) in machine readable format.
