# Supplementary material for: Bubble Point Measurements of cis-1,1,1,4,4,4-Hexafluorobutene [R-1336mzz(Z)] + trans-1,2-Dichloroethene [R-1130(E)] mixtures
Source: Int J Thermophys. Author manuscript; Available in PMC 2025 May 6. (PMC12053543; doi:10.1007/s10765-024-03388-2)
Supplement: Supp1 [file NIHMS2022140-supplement-Supp1.docx]

Supporting Information:

Bubble Point Measurements of *cis*-1,1,1,4,4,4-Hexafluorobutene [R-1336mzz(Z)] + *trans*-1,2-Dichloroethene [R-1130(E)] mixtures

Aaron J. Rowane[[1]](#footnote-2)

Stephanie L. Outcalt

National Institute of Standards and Technology**,** Material Measurement Laboratory, Applied Chemicals and Materials Division, 325 Broadway, Boulder, CO 80305-3337, U.S.A.

Details of the Bubble Point Apparatus

Figure S1 is a schematic of the bubble point instrument used in this work. The operating range of the apparatus is 265 K to 360 K, to pressures of 7 MPa. The instrument is the same as that described in our previous studies^1, 2^.


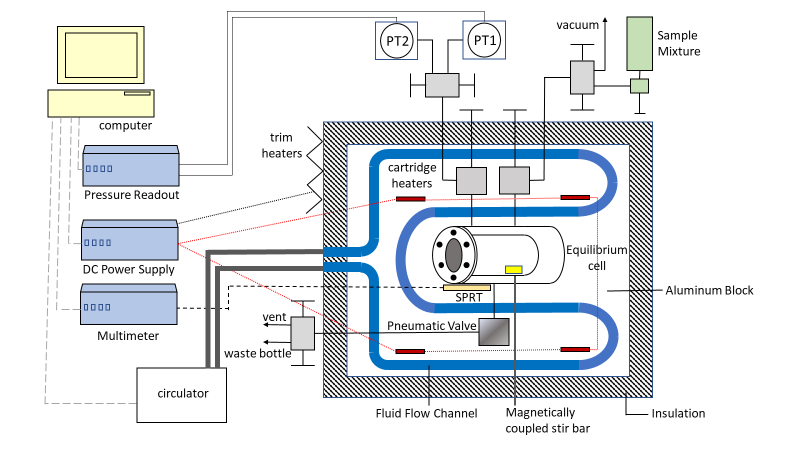


Figure S1. Schematic of the apparatus used to make the bubble point measurements.

The heart of the instrument is a cylindrical stainless steel cell with opposing sapphire windows on each end for visual access of the cell contents. The cell has an internal volume of approximately 30 mL. There are two ports at the top of the cell and one at the bottom. The valves closest to those ports are located inside the aluminum block to limit the volume of sample outside of the temperature-controlled environment. The additional volume of the system inside the aluminum block (excluding the equilibrium cell) is estimated to be no greater than 2.5 mL.

The temperature is measured with a standard platinum resistance thermometer (SPRT) situated in a thermowell along the side of the equilibrium cell. The aluminum block features internal flow channels for the circulation of cooling fluid and cut-outs containing cartridge heaters. Trim heaters are adhered to the exterior of the block and the block is surrounded by 5 cm of insulation. Temperature control of the system is fully automated incorporating a PID routine developed by Hust et al.^3^ Once the system has reached thermal equilibrium at a given temperature set point, the stability of the temperature is typically ± 5 mK.

The bubble point pressure is measured with one of two oscillating quartz crystal pressure transducers (PT1 and PT2 in Figure S1) connected to the system through one of the ports at the top of the cell. The range of the first transducer is to 0.7 MPa and the second to 7 MPa. The manufacturers’ stated accuracy of the pressure transducers is 0.01% of full range. Hence, to reduce the uncertainty in our measurements the lower range transducer is used for bubble point pressures below 0.7 MPa, then isolated from the system for higher pressure measurements, where the high range transducer is used. Each transducer is separately housed in its own temperature-controlled block which is maintained at 313 K.

The second port at the top of the cell connects to valving that facilitates filling and evacuating the system. The port at the bottom of the cell connects to a computer controlled, normally closed, pneumatic valve. During measurements, as the temperature of the system is increased, the bubble in the top of the cell disappears due to expansion of the sample. The pneumatic valve is used to remove small amounts of liquid from the cell to maintain a small bubble. Upon completion of a set of measurements, the pneumatic valve is controlled to remain open so that the cell contents can be cryo-pumped into a waste bottle.

Calculation of Incomplete Evacuation of Samples

Calculation of the air contribution to the pressure uncertainty of bubble point measurements.

●If you have 50 mtorr of air remaining in the sample bottle after evacuation.

●50 mtorr of pressure is equivalent to 0.006665 kPa at 295 K

●From REFPROP version 10.0 (hereafter REFPROP), the density of air at 0.006665 kPa and 295 K is 0.0000000787 g/ml.

So, if all the air from a sample bottle gets loaded into the bubble point cell of volume ~ 30 ml, the calculation is

0.0000000787 g/ml ● 30 ml = 0.000002361 grams of air in the cell.

Assuming all the air is compressed into the bubble at the top of the cell with the bubble having an estimated volume of 1 ml. From REFPROP, 0.000002361 grams of air in 1 ml at 295 K has a pressure of 0.20 kPa. This is therefore the dissolved air uncertainty in the pressure at 295 K. If this calculation is done at 360 K the pressure is approximately 0.24 kPa and at 265 K the pressure is 0.18 kPa. This is assuming that all of the air in the sample bottle gets transferred into the cell upon loading. This is a very unlikely scenario as the bottle is inverted prior to and during loading. So, 0.1 kPa was chosen as the uncertainty contribution for incomplete vacuum.

The Perturbed-Chain Statistical Associating Theory Equation of State

Thermodynamic property calculations using the PC-SAFT EOS start with the residual Helmholtz free energy, *ã*^res^, defined by equation 1,

$\tilde{a}^{res}=\tilde{a}^{hc}+\tilde{a}^{disp}$ (1)

where *ã*^hc^, is a hard-chain reference contribution, and *ã*^disp^, is a dispersion contribution. The hard chain contribution is defined by equation 2,

$\tilde{a}^{hc}=\bar{m}\tilde{a}^{hs}-\sum_{i} x_{i}(m_{i}-1)\ln g_{jj}^{hs}\left(\sigma_{jj}\right)$ (2)

where $\bar{m}$is the mean segment diameter given by equation 3, *ã*^hs^ is the Helmholtz free energy of a hard sphere given by equation 4, *x*_i_ is the molar composition of component *i*, and $g_{jj}^{hs}(\sigma_{jj})$ is the radial distribution function for a hard sphere given by equation 5.

$\bar{m}=\sum_{i} x_{i}m_{i}$ (3)

$\tilde{a}^{hs}=\frac{1}{\zeta_{0}}\left[\frac{3\zeta_{1}\zeta_{2}}{\left(1-\zeta_{3}\right)}+\frac{\zeta_{2}^{3}}{\zeta_{3}\left(1-\zeta_{3}\right)^{2}}+\left(\frac{\zeta_{2}^{3}}{\zeta_{3}^{2}}-\zeta_{0}\right)\ln\left(1-\zeta_{3}\right)\right]$ (4)

$g_{ij}^{hs}=\frac{1}{\left(1-\zeta_{3}\right)}+\left(\frac{d_{i}d_{j}}{d_{i}+d_{j}}\right)\frac{3\zeta_{2}}{\left(1-\zeta_{3}\right)^{2}}+\left(\frac{d_{i}d_{j}}{d_{i}+d_{j}}\right)^{2}\frac{2\zeta_{2}^{2}}{\left(1-\zeta_{3}\right)^{3}}$ (5)

$\zeta_{n}$ is defined by,

$\zeta_{n}=\frac{\pi}{6}\rho\sum_{i} x_{i}m_{i}d_{i}^{n}\begin{matrix} & & n\in\left\{0,1,2,3\right\} \end{matrix}$ (6)

Where *ρ* is the number density of molecules and *d*_i_, the temperature-dependent segment diameter is defined by,

$d_{i}=\sigma_{i}\left[1-0.12\exp\left(-3\frac{\varepsilon_{i}}{k_{b}T}\right)\right]$ (7)

The dispersion contribution to the residual Helmholtz free energy is defined by,

$\tilde{a}^{disp}=-2\pi\rho I_{1}\left(\eta,\bar{m}\right)\overline{m^{2}\varepsilon\sigma^{3}}-\pi\rho\bar{m}C_{1}I_{2}\left(\eta,\bar{m}\right)\overline{m^{2}\varepsilon^{2}\sigma^{3}}$ (8)

Where:

$\overline{m^{2}\varepsilon\sigma^{3}}=\sum_{i} \sum_{j} x_{i}x_{j}m_{i}m_{j}\left(\frac{\varepsilon_{ij}}{kT}\right)\sigma_{ij}^{3}$ (9)

$\overline{m^{2}\varepsilon^{2}\sigma^{3}}=\sum_{i} \sum_{j} x_{i}x_{j}m_{i}m_{j}\left(\frac{\varepsilon_{ij}}{kT}\right)^{2}\sigma_{ij}^{3}$ (10)

Expressions for $I_{1}\left(\eta,\bar{m}\right)$, $I_{2}\left(\eta,\bar{m}\right)$, and *C*_1_ are functions of *η*, the packing fraction, and $\bar{m}$which are omitted here and can be found elsewhere^4^. Mixture parameters *ε*_ij_ and *σ*_ij_ are defined by conventional mixing rules,

$\sigma_{ij}=\frac{\left(\sigma_{i}+\sigma_{j}\right)}{2}$ (11)

$\varepsilon_{ij}=\left(1-k_{ij}\right)\sqrt{\varepsilon_{i}\varepsilon_{j}}$ (12)

Where *k*_ij_ is the binary interaction parameter determined from the best fit of the data.

NMR Sample Purity Analysis

A degassed sample of R1130(E) (trans-1,2-dichloroethene) was presented in a stainless-steel sample cylinder (Swagelok 304L-HDF4-2250). To prepare an NMR sample of neat R-1130(E), a simple manifold with a central T was assembled. The glass NMR tube and the sample cylinder were connected opposite of each other on the central T. The vacuum line was connected to the third position on the T (Fig. S2). The manifold and the glass sample tube were evacuated for about 30 minutes while the fluid in the sample cylinder was warmed with a heat lamp. At this point, the sample cylinder was pleasantly warm to the touch (about 35 °C). Then the bottom half of the glass NMR tube was immersed in liquid nitrogen, the valve to the vacuum line was closed, and the valve on the sample cylinder was opened. Within a few seconds, the glass sample tube was about half full of liquid, so the valve on the sample cylinder was closed. Then the PTFE valve on the top of the glass NMR tube was closed and the NMR tube was removed from the manifold. The NMR tube was immediately transferred into the benchtop NMR and a cardboard box was placed over the sample bore to exclude ambient light. The sample experienced less than 5 minutes of ambient light exposure during this process.

The NMR sample went through a 5 min shimming routine, after which the linewidth (at 50 %) was 0.709 Hz. Test spectra for ^1^H and ^13^C were collected (about 15 minutes total). Then, a ^1^H spectrum was collected by averaging 1064 scans (90° pulse, acquisition time of 3.2 s, 16k data points, and pulse repetition time of 4.0 s). This spectrum took about an hour to collect. The spectrum was phased manually, after which automated baseline flattening was applied. Line broadening of 0.2 Hz resulted in a signal to noise ratio of 6.1 × 10^3^ for the main sample peak.

The spectrum of R-1130(E) consists of a singlet peak at 6.33 ppm with characteristic carbon satellites (*J*_C-H_ ~200 Hz, each satellite is a doublet). The spectrum was referenced to trace acetone (2.05 ppm) in a separately prepared sample (see below). A multitude of impurity peaks appeared in the range of 0.7 ppm to 3.0 ppm. The total intensity of the impurity peaks was 0.9 %. **Thus, the sample of R-1130(E) is (99.10 ± 0.10) % pure by ^1^H NMR.**

There were two general concerns about this purity analysis. First, this sample has some chemical instability, and it is sensitive to light. The question was, did the impurities come from the stainless-steel sample container or did they form during the sample handling and analysis. Two additional spectra were collected to check sample stability. A second spectrum was collected after the sample had been in the spectrometer for 4 days. Then the sample was removed from the spectrometer and left on the benchtop in 2-1107 (with all the lights on) for 30 minutes before a third spectrum was collected. The second and third spectra showed purities of 99.25 % and 99.22%, respectively. Thus, sample decomposition was ruled out. The second concern was that the impurity peaks looked suspiciously like a hydrocarbon oil. The question was, did they come from the sample itself or did they come from the transfer line between the stainless-steel sample container and the glass NMR tube. The transfer line and the NMR sample cell were rinsed with acetone before taking a second sample from the stainless-steel sample container. A ^1^H spectrum of this sample showed a purity of 99.17 % with the same suite of impurities as the first sample. This spectrum also had a small acetone peak (excluded from the purity value listed above), which was used to reference the spectral peaks. In any case, the lack of a change in the spectrum after rinsing the transfer line and NMR tube suggests that the contamination did not occur during sample preparation.


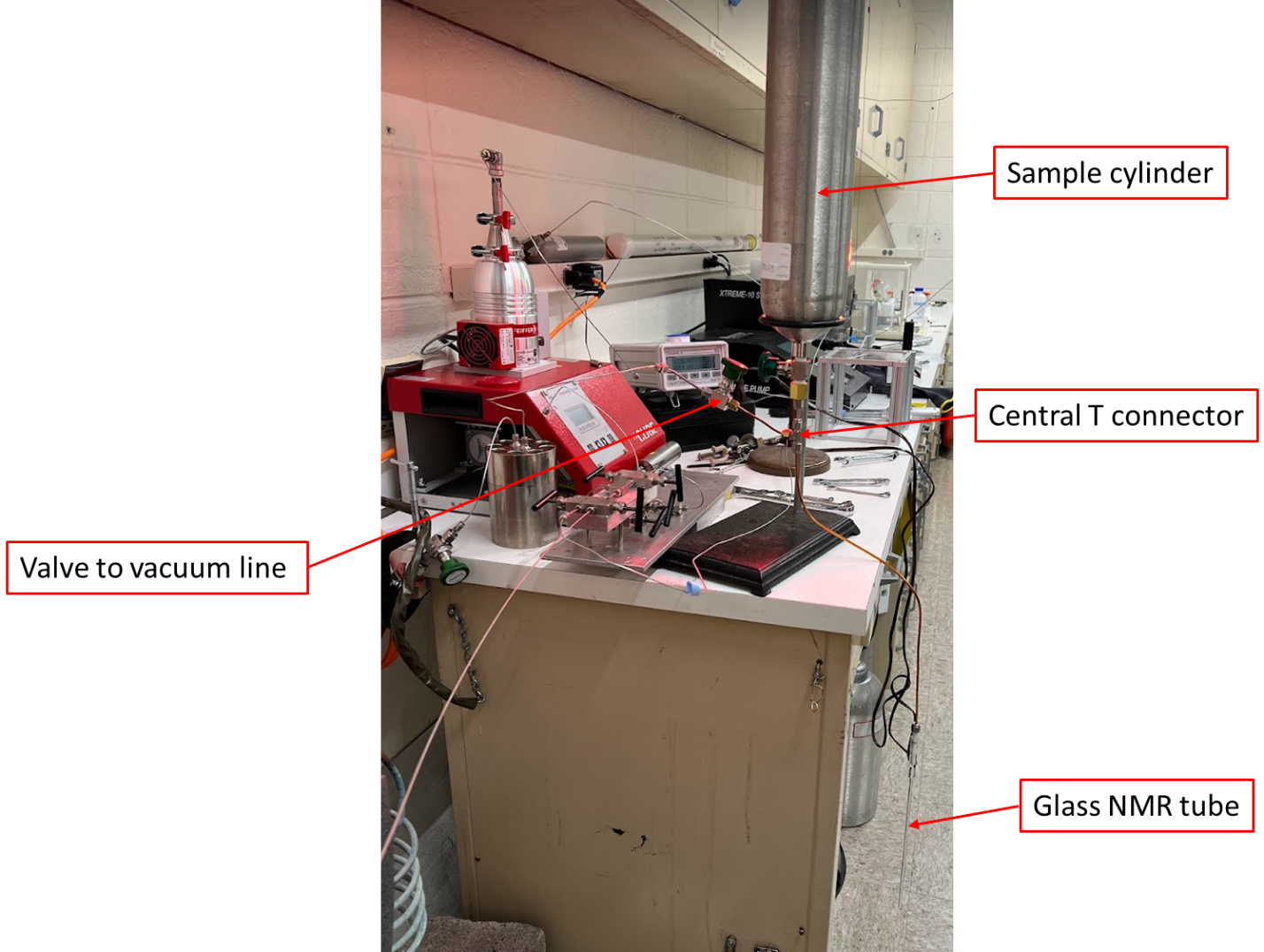


Figure S2. NMR filling setup.

**References:**

(1) Outcalt, S. L.; Rowane, A. J. Bubble Point Measurements of Mixtures of HFO and HFC Refrigerants. *J Chem Eng Data* **2021**, *66* (12), 4670-4683. DOI: <https://doi.org/10.1021/acs.jced.1c00654>.

(2) Outcalt, S. L.; Rowane, A. J. Bubble Point Measurements of Three Binary Mixtures of Refrigerants: R-32/1234yf, R-32/1234ze(E), and R-1132a/1234yf. *J Chem Eng Data* **2022**, *67* (4), 932-940. DOI: <https://doi.org/10.1021/acs.jced.1c00871>.

(3) Hust, J. G.; Filla, J.; Smith, D. R. A Modified Digital PID Temperature Controller for Thermal Properties Measurements. *J. Thermal Insulation* **1987**, *11*, 102 - 107.

(4) Gross, J.; Sadowski, G. Perturbed-Chain SAFT: An Equation of State Based on a Perturbation Theory for Chain Molecules. *Ind Eng Chem Res* **2001**, *40* (4), 1244-1260. DOI: <https://doi.org/10.1021/ie0003887>.

1. Corresponding author. [↑](#footnote-ref-2)
